# Delayed Chylothorax After Endometrial Carcinoma: A Case of Confirmed Thoracic Duct Leak Successfully Managed Conservatively

**DOI:** 10.7759/cureus.108336

**Published:** 2026-05-06

**Authors:** Swetha Naveen, Thangaswamy Dhanasekar

**Affiliations:** 1 Respiratory Medicine, Sri Ramachandra Institute of Higher Education and Research, Chennai, IND; 2 Pulmonology, Sri Ramachandra Institute of Higher Education and Research, Chennai, IND

**Keywords:** carcinoma endometrium, chylothorax, lymphatic leak, non-traumatic chylothorax, pleural effusion

## Abstract

Chylothorax is an uncommon cause of pleural effusion resulting from the disruption or obstruction of the thoracic duct or its tributaries. Malignancy is a major non-traumatic cause, most commonly lymphoma, while involvement of gynecologic malignancies is exceedingly uncommon and largely limited to isolated case reports.

We report the case of a 61-year-old woman with a prior history of poorly differentiated endometrial carcinoma treated in 2022 with total abdominal hysterectomy, bilateral salpingo-oophorectomy, and adjuvant brachytherapy, who presented three years later with progressive dyspnea, cough, anorexia, and weight loss. Imaging revealed recurrent left-sided pleural effusion. Diagnostic thoracentesis yielded milky fluid, and pleural fluid analysis demonstrated markedly elevated triglyceride levels with relatively low cholesterol, consistent with chylothorax, with negative microbiology and cytology for malignant cells.

Intranodal lymphangiography confirmed an active thoracic duct leak into the left pleural cavity. Despite this, conservative management was pursued due to the patient’s stable clinical status and preference to defer intervention, consisting of therapeutic thoracentesis and dietary modification with a low-fat diet supplemented by medium-chain triglycerides. This resulted in significant clinical and radiological resolution.

This case highlights a delayed presentation of chylothorax following endometrial carcinoma and emphasizes a systematic diagnostic approach with individualized management, demonstrating successful conservative resolution despite a confirmed thoracic duct leak.

## Introduction

Chylothorax is an uncommon but clinically significant cause of pleural effusion, resulting from disruption or obstruction of the thoracic duct and leading to accumulation of chyle within the pleural space. Although uncommon, it carries substantial morbidity due to the continuous loss of lymphatic fluid rich in triglycerides, proteins, and lymphocytes, which may result in malnutrition, immunosuppression, and metabolic disturbances if not promptly recognized and managed [1,2].

The etiology of chylothorax is broadly classified into traumatic and non-traumatic causes, with malignancy being the most common non-traumatic etiology, most frequently due to lymphoma. This is attributed to lymphatic obstruction or infiltration by malignant disease. Chylothorax has been less frequently reported secondary to solid tumors and has been related to mechanisms including lymphatic obstruction, tumor invasion, or disruption of lymphatic flow [3-5].

Diagnosis is primarily established by pleural fluid analysis, with triglyceride levels exceeding 110 mg/dL being diagnostic. Imaging modalities such as computed tomography (CT) and lymphangiography are useful in identifying the underlying cause and localizing the site of lymphatic disruption [6-8]. Clinically, patients often present with progressive dyspnea and recurrent pleural effusion, and the condition may be misdiagnosed as malignant or parapneumonic effusion, particularly in those with a prior history of cancer.

Endometrial carcinoma is one of the most common gynecologic malignancies and is usually diagnosed at an early stage because of its characteristic clinical presentation [9]. Chylothorax associated with gynecologic malignancies, particularly endometrial carcinoma, is exceedingly rare and has been described only in a limited number of case reports [10] and may reflect atypical lymphatic spread or delayed effects related to prior treatment, including treatment-related lymphatic injury or altered lymphatic flow dynamics.

We present a case of unilateral chylothorax occurring three years after treatment for endometrial carcinoma, highlighting an unusual delayed temporal association in the absence of radiological evidence of recurrence. This case underscores the diagnostic challenge in distinguishing between occult malignancy and non-malignant lymphatic disruption and demonstrates that a conservative approach may be successful in carefully selected patients despite lymphangiographic evidence of thoracic duct leakage.

## Case presentation

A 61-year-old woman presented with complaints of left-sided chest pain of one-week duration. The pain was insidious in onset, gradually progressive, and aggravated by lying on the left side and deep inspiration. There was no significant dyspnea at presentation.

She had a prior history of poorly differentiated (grade 3) endometrial carcinoma diagnosed three years earlier; however, complete staging and nodal status were not available despite efforts to retrieve these details from prior medical records. She had undergone a total abdominal hysterectomy with bilateral salpingo-oophorectomy in 2022, followed by adjuvant brachytherapy. A CT scan of the chest performed at that time showed no evidence of pulmonary metastasis. Her comorbidities included type 2 diabetes mellitus (HbA1c: 6.7%), systemic hypertension, and obesity (body mass index: 36.6 kg/m²).

On physical examination, absent breath sounds and stony dullness to percussion were noted over the left lower lung fields, suggestive of pleural effusion.

**Figure 1:**
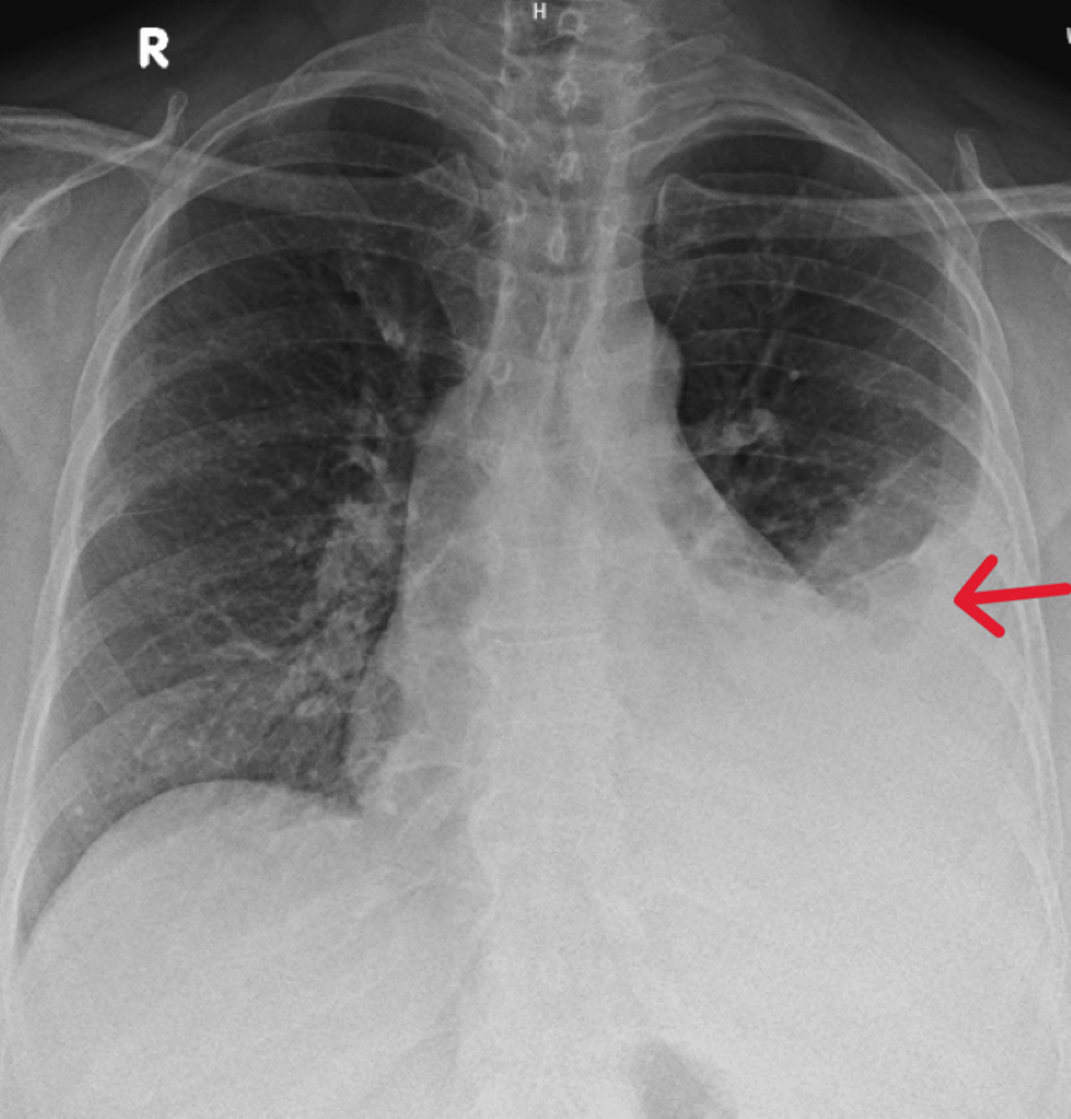
Initial chest radiograph demonstrating homogeneous opacification of the left mid and lower lung zones with blunting of the left costophrenic angle (arrow), consistent with left-sided pleural effusion.

A chest radiograph revealed homogeneous opacification of the left mid and lower lung zones with blunting of the left costophrenic angle (Figure 1). 

Electrocardiogram and 2D echocardiography were unremarkable. Routine laboratory investigations, including complete blood count, renal function tests, and liver function tests, were within normal limits.

Diagnostic thoracentesis yielded approximately 500 mL of milky fluid. Pleural fluid analysis (Table 1) demonstrated an exudative effusion with elevated protein levels, consistent with Light’s criteria. Cytological examination was negative for malignant cells, and microbiological investigations, including culture, Gram stain, and testing for tuberculosis (GeneXpert and ADA), were unremarkable. The pleural fluid glucose level was 164 mg/dL, making empyema less likely.

**Table 1:** Pleural fluid analysis demonstrating markedly elevated triglyceride levels with relatively low cholesterol, a pattern diagnostic of chylothorax. ADA: adenosine deaminase, LDH: lactate dehydrogenase, MTB: *Mycobacterium tuberculosis.*

Parameter	Value	Reference range
Appearance	Milky	Clear/straw-colored
Triglycerides	2,153 mg/dL	<110 mg/dL
Cholesterol	83 mg/dL	<200 mg/dL
Protein	6.05 g/dL	<3 g/dL
LDH	116 U/L	Serum-dependent
Glucose	164 mg/dL	70-140 mg/dL
Total cell count	600 cells/mm³	<500
Differential	Lymphocyte-predominant	-
ADA	6 U/L	<40 U/L
Cytology	Negative	-
Microbiology (culture, Gram stain)	Negative	-
Truenat MTB	Not detected	-

Notably, triglyceride levels were markedly elevated (2,153 mg/dL), well above the diagnostic threshold of 110 mg/dL, with relatively low cholesterol levels, confirming the diagnosis of chylothorax [6]. It is to be noted that there was no history of recent trauma or thoracic surgery.

The patient’s weight loss raised concern for possible occult malignancy recurrence. However, contrast-enhanced CT of the chest showed no evidence of pulmonary metastasis or mediastinal lymphadenopathy, and pleural fluid cytology was negative for malignant cells. Infectious causes, including tuberculosis, were excluded based on negative microbiological studies and low ADA levels. Cardiac evaluation was unremarkable. PET CT was not performed, as no clinical, cytological, or radiological evidence of recurrence was identified; however, the presence of occult or microscopic disease cannot be entirely excluded.

**Figure 2:**
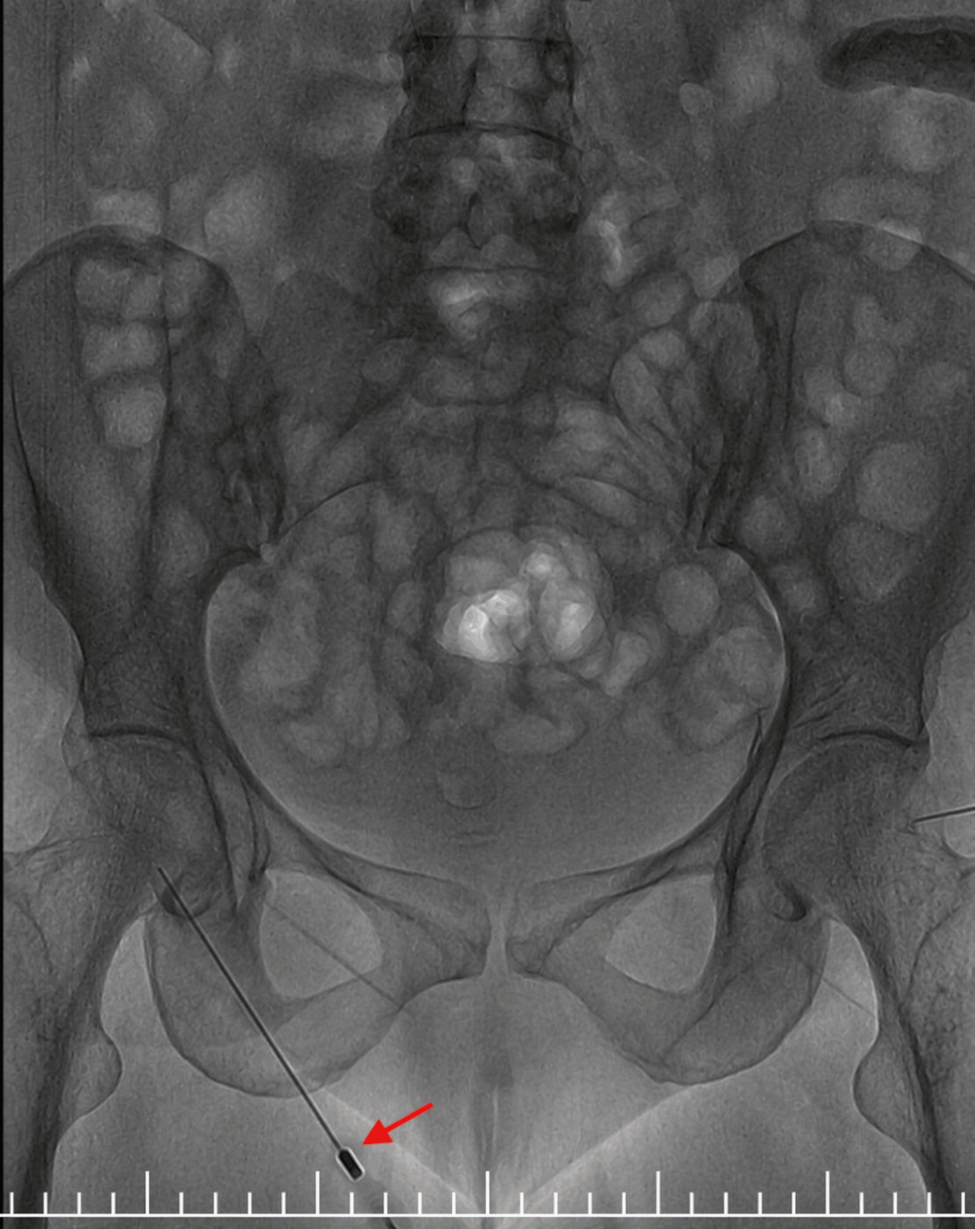
Intranodal lymphangiography demonstrating cannulation of the inguinal lymph node (arrow) with contrast administration. Subsequent imaging revealed extravasation of contrast from the thoracic duct at the T8-T9 level into the left pleural cavity, consistent with an active lymphatic leak.

**Figure 3:**
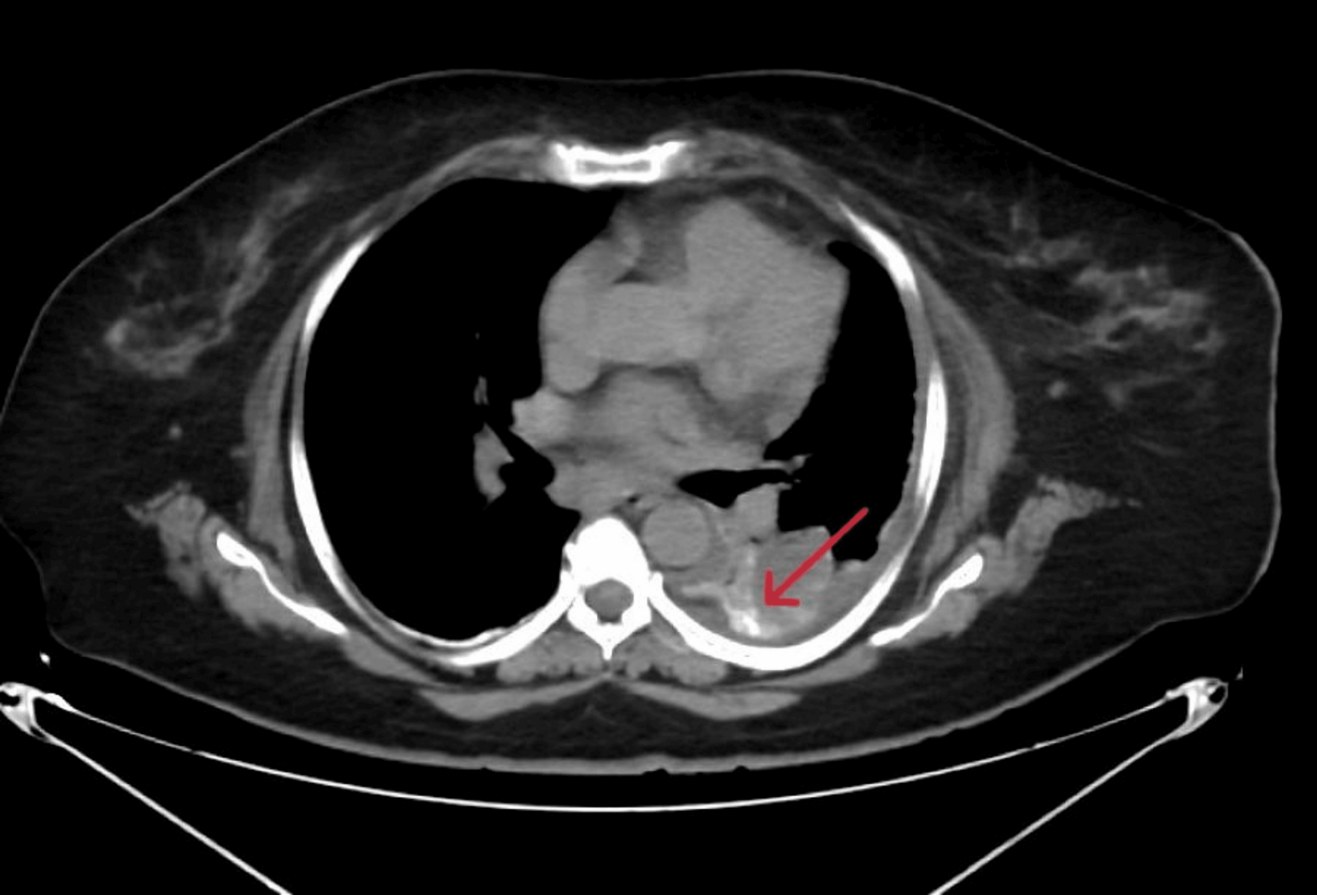
CT of the chest demonstrating contrast accumulation within the left pleural cavity (arrow), representing extravasation following lymphangiography and confirming a thoracic duct leak.

The patient was initially evaluated on an outpatient basis in October and, due to persistent symptoms, was admitted in January for further management at her subsequent visit, requiring a hospital stay of seven days. Given the persistence of chylous effusion, intranodal lymphangiography (Figure 2) was performed to evaluate the lymphatic system. Bilateral inguinal lymph nodes were cannulated, and lipiodol contrast was administered. The study demonstrated a paucity of para-aortic and lumbar lymphatic channels with non-visualization of the cisterna chyli. Notably, contrast extravasation from the thoracic duct at the T8-T9 vertebral level into the left pleural cavity was observed, confirming an active lymphatic leak. Additionally, normal drainage of the thoracic duct into the left subclavian vein was not visualized. Subsequent imaging showed accumulation of lipiodol within the left pleural space, further supporting the diagnosis (Figure 3).

The patient was managed conservatively with repeated therapeutic thoracentesis and dietary modification, including a low-fat diet supplemented with medium-chain triglycerides to reduce lymphatic flow. Thoracic duct embolization was advised; however, the patient deferred intervention.

Thus, despite lymphangiographic evidence of a thoracic duct leak, conservative management was considered appropriate, given the patient’s clinical stability, progressively decreasing chyle output, absence of significant nutritional or immunological compromise, and patient preference to avoid invasive intervention. Close clinical and biochemical follow-up was ensured.

**Figure 4:**
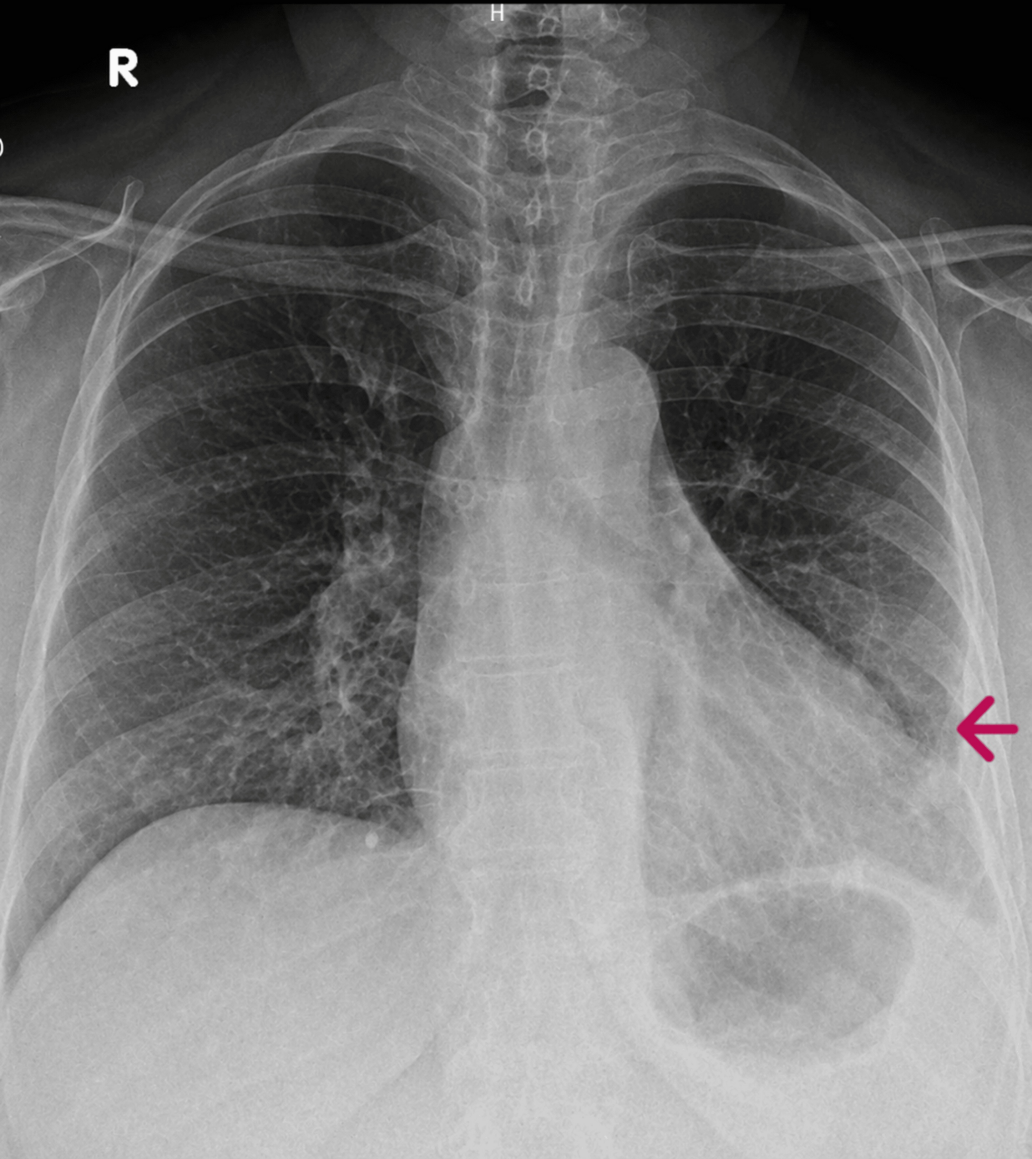
Follow-up chest radiograph performed four months later demonstrating significant resolution of the left-sided pleural effusion following conservative management.

Follow-up imaging performed four months later demonstrated significant resolution of the pleural effusion (Figure 4). Repeat pleural fluid analysis showed a marked reduction in triglyceride levels to 15 mg/dL, consistent with resolution.

## Discussion

Chylothorax results from the disruption or obstruction of the thoracic duct, leading to the accumulation of chyle in the pleural space and potential metabolic and immunological complications if not appropriately managed [1,2]. Among non-traumatic causes, malignancy is the most common etiology, most frequently due to lymphoma. This is attributed to lymphatic obstruction, direct infiltration of the thoracic duct, or compression by enlarged lymph nodes [1,3,4].

In contrast, chylothorax associated with solid tumors is less commonly reported and is typically related to mediastinal lymph node involvement, tumor infiltration, or disruption of lymphatic drainage pathways [4,5]. Involvement of gynecologic malignancies, including endometrial carcinoma, is particularly rare and has been described mainly in isolated case reports. Reported cases include postoperative presentations and associations with chylous ascites [11]; however, delayed thoracic involvement years after treatment is rarely documented. A similar case has been reported in the literature, including one in the CHEST journal describing chylothorax following treatment for endometrial carcinoma, supporting the rarity of this presentation [10].

The mechanism of chylothorax in such cases remains uncertain, particularly in the absence of radiologically evident metastasis. Proposed explanations include subclinical lymphatic obstruction, delayed effects of prior surgery or radiotherapy, or altered lymphatic flow dynamics rather than direct tumor invasion.

In the present case, the diagnosis was established based on classical biochemical findings, including milky pleural fluid and markedly elevated triglyceride levels (2,153 mg/dL), exceeding the diagnostic threshold of 110 mg/dL [6]. Intranodal lymphangiography demonstrated contrast extravasation from the thoracic duct at the T8-T9 level, confirming an active lymphatic leak and providing a definitive anatomical basis for the effusion. This distinguishes the case from many reported instances in which the diagnosis remains presumptive [7]. The differential diagnosis included malignancy-related chylothorax, infectious causes (including tuberculosis), and traumatic or iatrogenic etiologies. Malignancy recurrence was considered; however, negative cytology, absence of radiological evidence of recurrence, and clinical stability made this unlikely. Infectious causes were excluded based on negative microbiological evaluation, and there was no history of recent trauma or surgery.

Conservative management remains the first-line approach in chylothorax and is aimed at reducing chyle flow and allowing spontaneous closure of the leak. Dietary modification plays a central role, as medium-chain triglycerides are absorbed directly into the portal circulation, thereby bypassing the lymphatic system and reducing thoracic duct flow [1,2]. In the present case, this approach resulted in significant clinical and radiological improvement despite confirmed ductal disruption. The decision to pursue conservative management despite a documented leak was supported by the patient’s clinical stability, low and progressively decreasing chyle output, absence of metabolic or immunological compromise, and patient preference.

The reported success of conservative management varies widely in the literature, with rates ranging from approximately 15% to 94% in non-traumatic chylothorax, reflecting heterogeneity in patient populations and disease severity [3]. In addition to dietary measures, lymphangiography itself may contribute to resolution through a therapeutic effect. Lipiodol contrast can induce a localized inflammatory response leading to embolization of the leak site, thereby promoting closure [7,8], which may have contributed to the favorable outcome observed in this patient. The lymphangiographic findings may also reflect underlying alterations in lymphatic flow or obstruction.

Although conservative management is effective in selected cases, surgical or interventional management is generally considered in patients with high-output chylothorax (commonly defined as >1,000 mL/day in adults), persistent chyle leak beyond 1-2 weeks despite conservative therapy, or those who develop nutritional, metabolic, or immunological complications. Additional indications include failure of conservative management and progressive clinical deterioration, in which case thoracic duct embolization or surgical ligation may be required [3,5].

This case is clinically significant as it demonstrates a delayed presentation of chylothorax occurring three years after treatment for endometrial carcinoma in the absence of clear evidence of recurrence. However, complete oncologic staging details were not available, and PET CT was not performed; therefore, while no evidence of recurrence was identified, the presence of occult disease cannot be entirely excluded. The association with prior endometrial carcinoma should be interpreted as temporal rather than causal. The case also highlights the diagnostic value of lymphangiography in confirming thoracic duct leakage and suggests that conservative management may be successful in carefully selected patients, even in the presence of a documented structural defect.

## Conclusions

This case documents a delayed-onset unilateral chylothorax presenting three years after treatment for poorly differentiated endometrial carcinoma, in the absence of radiological or cytological evidence of recurrence, although the association with prior endometrial carcinoma should be interpreted as temporal rather than causal. The diagnosis was established with high certainty through markedly elevated pleural fluid triglyceride levels and intranodal lymphangiographic confirmation of a thoracic duct leak at the T8-T9 level. Notably, despite objective demonstration of a structural lymphatic disruption, the patient experienced complete clinical and radiological resolution with conservative management alone, including pleural drainage and dietary modification. These findings suggest that a conservative approach may be successful in carefully selected patients, even when a thoracic duct leak is documented.

## References

[1] Bhatnagar M, Fisher A, Ramsaroop S, Carter A, Pippard B (2024). Chylothorax: pathophysiology, diagnosis, and management-a comprehensive review. J Thorac Dis.

[2] Habas E, Farfar K, Errayes A (2023). Updates on the pathophysiology and therapies of chylous pleural effusion: a narrative review. Yemeni J Med Sci.

[3] Porcel JM, Bielsa S, Civit C (2023). Clinical characteristics of chylothorax: results from the International Collaborative Effusion database. ERJ Open Res.

[4] Riley LE, Ataya A (2019). Clinical approach and review of causes of a chylothorax. Respir Med.

[5] McGrath EE, Blades Z, Anderson PB (2010). Chylothorax: aetiology, diagnosis and therapeutic options. Respir Med.

[6] Staats BA, Ellefson RD, Budahn LL, Dines DE, Prakash UB, Offord K (1980). The lipoprotein profile of chylous and nonchylous pleural effusions. Mayo Clin Proc.

[7] Itkin M, Kucharczuk JC, Kwak A, Trerotola SO, Kaiser LR (2010). Nonoperative thoracic duct embolization for traumatic thoracic duct leak: experience in 109 patients. J Thorac Cardiovasc Surg.

[8] An R, Xia S, Sun Y, Chang K, Li Y, Shen W (2021). New application of direct lymphangiography in the diagnosis and treatment of chylothorax after lung cancer surgery: a case series. Ann Palliat Med.

[9] Bassette E, Ducie JA (2024). Endometrial cancer in reproductive-aged females: etiology and pathogenesis. Biomedicines.

[10] Ventura A, Lopez R, Rutazaana D, Gupta A, Pourshahid S (2018). Chylothorax presenting in a patient with endometrial cancer. Chest.

[11] Tani K, Ogushi F, Sone S, Kagawa N, Ogura T (1988). Chylothorax and chylous ascites in a patient with uterine cancer. Jpn J Clin Oncol.

